# Recent Advances in Understanding the Molecular Mechanisms of SGLT2 Inhibitors in Atrial Remodeling

**DOI:** 10.3390/cimb46090571

**Published:** 2024-08-31

**Authors:** Ioan-Alexandru Minciună, Raluca Tomoaia, Dragos Mihăilă, Gabriel Cismaru, Mihai Puiu, Radu Roșu, Gelu Simu, Florina Frîngu, Diana Andrada Irimie, Bogdan Caloian, Dumitru Zdrenghea, Dana Pop

**Affiliations:** 1 5th Department of Internal Medicine, Faculty of Medicine, “Iuliu Hațieganu” University of Medicine and Pharmacy, 400347 Cluj-Napoca, Romania; minciuna.ioan.alexandru@elearn.umfcluj.ro (I.-A.M.); marius.drag.mihaila@elearn.umfcluj.ro (D.M.); cismaru.gabriel@elearn.umfcluj.ro (G.C.); rosu.radu1053@elearn.umfcluj.ro (R.R.); florina.fringu@elearn.umfcluj.ro (F.F.); gurzau.diana@elearn.umfcluj.ro (D.A.I.); bogdan912@elearn.umfcluj.ro (B.C.); dzdrenghea@elearn.umfcluj.ro (D.Z.); dana.pop@umfcluj.ro (D.P.); 2 Cardiology Department, Rehabilitation Hospital, 400066 Cluj-Napoca, Romania; puiu.mihai@yahoo.com

**Keywords:** atrial remodeling, SGLT2 inhibitors, atrial fibrillation, atrial cardiomyopathy, heart failure

## Abstract

Atrial cardiomyopathy and remodeling play pivotal roles in the development of atrial fibrillation (AF) and heart failure (HF), involving complex changes in atrial structure and function. These changes facilitate the progression of AF and HF by creating a dynamic interplay between mechanical stress and electrical disturbances in the heart. Sodium–glucose cotransporter 2 inhibitors (SGLT2is), initially developed for the management of type 2 diabetes, have demonstrated promising cardiovascular benefits, being currently one of the cornerstone treatments in HF management. Despite recent data from randomized clinical trials indicating that SGLT2is may significantly influence atrial remodeling, their overall effectiveness in this context is still under debate. Given the emerging evidence, this review examines the molecular mechanisms through which SGLT2is exert their effects on atrial remodeling, aiming to clarify their potential benefits and limitations. By exploring these mechanisms, this review aims to provide insights into how SGLT2is can be integrated into strategies for preventing the progression of atrial remodeling and HF, as well as the development of AF.

## 1. Introduction

Atrial cardiomyopathy and remodeling are key features that contribute to the development of atrial fibrillation (AF) and heart failure (HF). While initially adaptative, these changes encompass a range of pathophysiological alterations in atrial structure and mechanical and electrical function [1,2,3,4]. Conversely, atrial remodeling can be triggered by various stimuli, including sustained high atrial rates and volume or pressure overload. Persistently high atrial rates, such as those observed in tachyarrhythmias and AF, induce alterations in atrial tissue due to continuous electrical and mechanical stress. This results in structural and functional changes in the atrial myocardium, contributing to the remodeling process. Also, volume and pressure overload, resulting from conditions such as mitral valve disease or HF, play a significant role in atrial remodeling by inducing atrial hypertrophy and changes in atrial mechanics. These processes collectively drive the progression of AF and HF, highlighting the complex interplay between mechanical stress and electrical disturbances in atrial remodeling [5,6].

Current therapies targeting cardiac remodeling in HF and AF face several challenges, including suboptimal efficacy and the considerable risk of adverse effects [5]. Originally developed for the management of type 2 diabetes, sodium–glucose cotransporter 2 inhibitors (SGLT2is) are currently recognized for their substantial cardiovascular protective effects in addition to their role in glycemic control [4,7]. Recent large-scale randomized clinical trials—including DEFINE-HF [8], PRESERVED-HF [9], EMPA-REG OUTCOME [10], DAPA-HF [11], DELIVER [12], EMPEROR-REDUCED [13], EMPEROR-PRESERVED [14], and EMPULSE [15]—have confirmed the cardiovascular benefits of SGLT2is. As a result, the latest European and American HF guidelines [16,17,18,19] now recommend the use of SGLT2is for both diabetic and non-diabetic HF patients, regardless of left ventricular ejection fraction.

Recent research published in the last five years has identified key mechanisms involved in atrial remodeling targeted by SGLT2is, including inflammation, oxidative stress, mitochondrial dysfunction, and the dysregulation of the autonomic nervous system [2,3,20,21]. These factors collectively contribute to disruptions in energy production and expenditure [22,23,24], lead to imbalances in atrial hormones and molecules, and cause impairments in sodium and calcium homeostasis [25,26]. Additionally, SGLT2is may influence processes such as fatty accumulation [23,24,26,27], apoptosis, and fibrosis [3,20,21,28], which are critical processes leading to irreversible structural changes in the atrium. SGLT2is exert their effects through several molecular pathways: they modulate critical inflammatory pathways and macrophage activity, reduce oxidative stress by lowering reactive oxygen species (ROS) levels, and enhance mitochondrial function. SGLT2is also address autonomic dysfunction by reducing sympathetic nerve activation by the lowering of circulating catecholamine levels. They inhibit fibroblast activation and alter fibrosis-associated signaling pathways. Additionally, SGLT2is improve myocardial energy efficiency by reducing epicardial adipose tissue and enhance calcium handling and mitochondrial function by lowering cytosolic sodium and calcium levels. A comprehensive understanding of these complex molecular and cellular mechanisms is crucial for developing future strategies and integrating novel therapeutic agents designed to effectively reduce cardiovascular events and mortality associated with atrial remodeling.

Despite recent randomized trials demonstrating that SGLT2is reduce the incidence of AF and prevent its recurrence following rhythm control therapy, the role of these agents on atrial remodeling remains a subject of debate. To address this issue, the present review examines the molecular mechanisms by which SGLT2is influence atrial remodeling. This review aims to enhance the understanding of the potential benefits and limitations of SGLT2is in the management of AF and HF, ultimately offering valuable insights for guiding future research and clinical practice.

## 2. Methods

To identify relevant publications, the authors conducted a search on PubMed using the following keyword combinations: “SGLT2” AND “atrial remodeling,” “SGLT2” AND “atrial fibrosis,” and “SGLT2” AND “atrial cardiomyopathy.” Articles addressing SGLT2 in relation to atrial remodeling, atrial fibrosis, or atrial cardiomyopathy were considered. Both human and animal studies were included. The initial search, performed in June 2024, yielded 51 studies published between 2017 and 2024, of which 2 were duplicates, 2 editorials, and 3 guidelines, resulting in a final selection of 44 articles (refer to Table 1 for the detailed selection process). A narrative review was subsequently created to summarize the findings. All selected studies, involving either human or animal subjects, adhered to research protocol approval requirements, with human studies obtaining informed consent where applicable.

## 3. Results and Discussions

### 3.1. Molecular Mechanisms of SGLT2is and Atrial Remodeling

**Figure 1:**
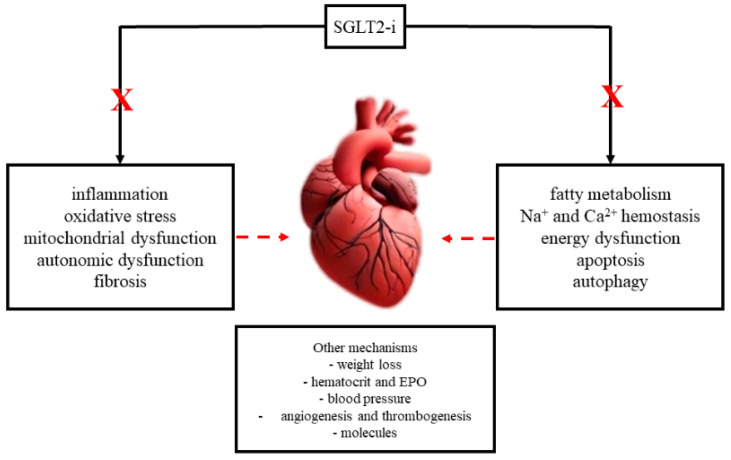
Mechanisms of SGLT2is on Atrial Remodeling.

Originally, it was believed that SGLT2is primarily exerted their effects through diuretic or natriuretic mechanisms. However, current evidence indicates that these effects are only temporary and not central to their action [46]. Instead, SGLT2is influence multiple systems, including the nervous, cardiovascular, and endocrine systems, resulting in intricate metabolic effects that go beyond simple glycemic control and contribute to mitigating myocardial remodeling and enhancing cardiovascular protection [48]. Figure 1 illustrates the various pathways through which SGLT2is exert their effects on atrial remodeling, including their impact on myocardial fibrosis, atrial dilation, electrophysiological properties, and molecular and cellular mechanisms.

#### 3.1.1. Inflammation

HF is often associated with elevated levels of circulating pro-inflammatory cytokines such as interleukin (IL)-1β, IL-6, IL-8, and tumor necrosis factor (TNF)-α, which contribute to adverse cardiac remodeling. Inflammation in HF is mediated by pattern recognition receptors (PRRs) like nucleotide-binding domain, leucine-rich–containing family, pyrin domain–containing-3 (NLRP3) and Toll-like receptor 4 (TLR4), which activate inflammatory pathways leading to cardiac hypertrophy and fibrosis. These cytokines promote immune cell activation and additional cytokine release, exacerbating the inflammatory and fibrotic processes in the heart [24]. The inflammatory biomarkers’ soluble suppression of tumorigenesis-2 (sST2) and galectin-3 are particularly noteworthy, as they provide prognostic information related to fibrosis and are more accurate than N-terminal pro-B-type natriuretic peptide (NT-proBNP) in assessing inflammation in HF [42].

SGLT2is have been shown to exert anti-inflammatory effects, which contribute to their cardioprotective benefits. Clinical meta-analyses indicate that SGLT2is reduce the levels of inflammatory markers such as IL-6, C-reactive protein, TNF-α, and monocyte chemotactic protein 1 (MCP-1), which are linked to systemic inflammation (28). Animal studies also support these findings, revealing that SGLT2is decrease cardiac inflammation by inhibiting inflammasome activation [19,20,26,38,39]. This action shifts macrophage polarization from the pro-inflammatory M1 phenotype to the anti-inflammatory M2 phenotype, thereby reducing inflammatory markers and improving cardiac function [28].

Myocardial infarction and ischemia–reperfusion injury trigger an inflammatory response that can worsen myocardial damage and remodeling. Macrophages play a crucial role in this context, with M1 macrophages driving inflammation and M2 macrophages aiding repair. SGLT2is, including empagliflozin and dapagliflozin, are noted for their ability to modulate macrophage activity, thereby reducing inflammation and enhancing cardiac repair processes, both in the atria and ventricles [28,39]. Atrial remodeling and AF are both associated with heightened levels of inflammation. Several preclinical [20,21,26,38,39,50] and clinical [3,4,54,60] studies have highlighted the role of SGLT2is in mitigating this inflammation, identifying it as a crucial mechanism underlying their cardioprotective effects.

#### 3.1.2. Oxidative Stress and Mitochondrial Dysfunction

Oxidative stress, characterized by an imbalance between ROS production and the cell’s antioxidant defenses, leads to significant cardiomyocyte damage. Excessive ROS, primarily generated by mitochondria, nicotinamide adenine dinucleotide phosphate oxidase (NADPH) oxidases, and nitric oxide synthase (NOS) uncoupling, can adversely affect proteins, lipids, and DNA [50]. Mitochondria play a crucial role in adenosine triphosphate (ATP) production and cellular homeostasis. They are particularly abundant in myocardial cells to support heart contraction, and their quality control involves processes such as fusion, fission, biogenesis, and mitophagy. Mitophagy, which can be receptor-dependent (e.g., BNIP3, BNIP3L) or ubiquitin-dependent (e.g., PINK/Parkin pathway) is essential for removing damaged mitochondria and maintaining cardiovascular health [28].

In diabetic models, mitochondrial dysfunction is notably prominent, contributing to the development of AF. Diabetic cardiomyocytes exhibit increased ROS production and mitochondrial membrane hyperpolarization, leading to mitochondrial damage and oxidative stress. This dysfunction impairs cardiac efficiency, promoting structural and electrical remodeling and increasing susceptibility to AF [8]. Both empagliflozin and dapagliflozin have been demonstrated to improve mitochondrial function and reduce ROS production [21,35]. In studies involving rats, empagliflozin enhanced mitochondrial function, improved electrophysiological abnormalities, and reduced atrial remodeling and AF inducibility [21].

The role of ROS in myocardial diseases such as AF and HF is significant. ROS production from sources like NADPH oxidases, mitochondria, and xanthine oxidases contributes to tissue damage, fibrosis, and impaired conduction, promoting atrial remodeling and arrhythmias. Mitochondrial dysfunction exacerbates these processes by reducing ATP synthesis and perpetuating the oxidative damage cycle. In a study by Nishinarita et al., the authors have shown that canagliflozin reduces oxidative stress and AF inducibility in animal models [38]. Similarly, research by Kondo et al. highlights that canagliflozin suppresses myocardial NADPH oxidase activity and improves NOS coupling, providing anti-inflammatory and anti-apoptotic benefits [41].

Adenosine monophosphate-activated protein kinase (AMPK), a key regulator of cellular energy, plays a role in mitochondrial health by promoting biogenesis, reducing ROS generation, and managing mitochondrial dynamics. In DM, AMPK activation is often impaired, but activators like empagliflozin can help manage cardiac issues and AF by enhancing AMPK-mediated mitochondrial dynamics [50]. This aligns with findings that show that AMPK activation through SGLT2is could protect against atrial remodeling in DM.

Overall, oxidative stress and mitochondrial dysfunction are pivotal in atrial remodeling and AF progression, in both diabetic and non-diabetic subjects. SGLT2is offer potential therapeutic benefits by improving mitochondrial function, reducing oxidative stress, and mitigating structural and electrical alterations in the atria [4].

#### 3.1.3. Autonomic Dysfunction

Autonomic dysfunction plays a pivotal role in the development of atrial remodeling with both sympathetic and parasympathetic imbalances contributing to increased AF risk. In patients with diabetes mellitus (DM), this dysfunction is characterized by heightened sympathetic and reduced parasympathetic activity, often observable through heart rate variability studies [35]. Autonomic remodeling in AF progresses through three stages: initial parasympathetic denervation, followed by sympathetic hyperactivation, and finally sympathetic denervation. Parasympathetic stimuli can lead to macro-re-entry phenomena, while sympathetic stimuli drive abnormal automaticity and trigger activity. In DM, autonomic dysfunction may precede the diagnosis, manifesting as impaired vagal responses and decreased acetylcholine release, which further influences AF by altering cardiac electrical activity [4].

SGLT2is have been shown to influence sympathetic nerve activation, which is crucial in cardiac remodeling. These inhibitors can reduce sympathetic nerve activation, as evidenced by lower tyrosine hydroxylase levels in the kidney and decreased circulating catecholamines [27]. The EMBODY trial, which investigated empagliflozin’s effects on cardiac nerve activity in type 2 DM and acute myocardial infarction patients, found that SGLT2is significantly enhance cardiac nerve activity while maintaining a favorable safety profile. Additionally, these inhibitors may lower blood pressure and sympathetic nerve activity, suggesting that SGLT2is effectively modulate sympathetic activation, although the exact mechanisms are still under investigation [28]. Clinical trials, including the EMBODY and EMPYREAN trials, demonstrate that SGLT2is such as empagliflozin can reduce sympathetic nerve activity and improve cardiac remodeling [3].

Interestingly, while increased heart rates typically exacerbate HF in patients with reduced ejection fraction, a recent case report by Zlahtic et al. proposes that in HF with preserved ejection fraction (HFpEF), higher heart rates combined with guideline-directed therapies, including SGLT2is, may positively affect atrial and ventricular structural and functional remodeling [58]. Thus, SGLT2is not only impact autonomic dysfunction but also hold potential benefits in mitigating adverse atrial and ventricular remodeling associated with various HF types.

#### 3.1.4. Fibrosis

Fibrosis plays a fundamental role in the pathophysiology of many cardiovascular diseases. It is characterized by the excessive accumulation of extracellular matrix (ECM) components, which leads to increased tissue stiffness and alters normal cardiac structure and function. The primary drivers of cardiac fibrosis are myofibroblasts, which are activated following myocardial injury. These cells, in conjunction with macrophages, lymphocytes, and mast cells, contribute to fibrosis by producing ECM proteins and promoting inflammation. Excessive ECM deposition further exacerbates cardiac dysfunction. The interplay between inflammation and fibrosis is particularly pronounced in conditions such as AF and DM. Inflammation-induced fibrosis involves abnormal ECM remodeling, which affects matrix composition and cardiac muscle function. In AF, atrial fibrosis is crucial for maintaining the condition, with epicardial cells differentiating into myofibroblasts. Angiotensin-II and oxidative stress further contribute to the arrhythmogenic remodeling of the atrium by activating the NF-κB pathway, promoting myofibroblast differentiation, and overexpressing ECM proteins, thereby increasing AF susceptibility in diabetic models [4].

Recent studies highlight the potential of SGLT2is in addressing myocardial fibrosis. Clinical research indicates that SGLT2is such as empagliflozin and dapagliflozin can improve cardiac function and reduce fibrosis, both in diabetic and non-diabetic patients. These drugs appear to influence fibrosis through the modulation of calcium and sodium levels in heart cells, reductions in inflammation, and alterations in various signaling pathways associated with fibrosis. Despite these promising findings, the precise mechanisms through which SGLT2is exert their cardioprotective effects are not yet fully understood [28].

For instance, dapagliflozin reduces myocardial fibrosis by inhibiting fibroblast activation and proliferation, primarily through the AMPK α-mediated suppression of TGF-β/Smad signaling. It also lowers oxidative stress and affects macrophage polarization, which helps improve cardiac remodeling and reduce fibrosis following myocardial infarction [3]. Similarly, in insulin-resistant female diabetic db/db mice, empagliflozin improved the expression of profibrotic proteins, such as serum/glucocorticoid-regulated kinase 1 (SGK1) and the epithelial sodium channel (ENaC), leading to reduced interstitial fibrosis in both atrial and ventricular myocardium [20]. Empagliflozin has been shown to directly influence human cardiac myofibroblast activity and ECM remodeling, indicating that it targets key profibrotic mechanisms involved in the progression of HF [33]. Additionally, Lin et al. demonstrated that empagliflozin could suppress cardiac fibrosis and endoplasmic reticulum stress while improving hemodynamics in a rat model of mitral regurgitation-induced HF [39]. Also, recent findings by Scatularo et al. suggest that endomyocardial fibrosis significantly contributes to the development of AF, and its reduction may be a crucial mechanism underlying the cardioprotective effects of SGLT2is [32,52].

Thereby, SGLT2is demonstrate significant potential in reducing myocardial fibrosis and atrial remodeling. By modulating various molecular and cellular pathways, these new agents can improve cardiac function, offering valuable cardioprotective benefits in conditions such as HF and AF.

#### 3.1.5. Myocardial Energy Metabolism

Fatty acids and glucose are the primary energy sources for myocardial metabolism. Under normal conditions, fatty acid metabolism, despite being more oxygen-demanding and less efficient for ATP production compared to glucose metabolism, serves as the main energy source. However, during pathological conditions such as AF, glycolysis becomes the predominant metabolic pathway. The role of AMPK is crucial in cardiac metabolism by enhancing fatty acid and glucose uptake to boost ATP production during energy shortages. During AF-induced metabolic stress, AMPK activation may help resist AF progression by restoring atrial calcium balance [24,35].

SGLT2is promote a metabolic shift from glucose to fatty acids, ketone bodies, and branched-chain amino acids, thereby improving myocardial energy efficiency. This metabolic switch has been observed across various species and was shown to enhance cardiac energetics in both in vitro and human studies [23,24,25,26,27]. Thirumatyam et al. demonstrated that SGLT2is increase lipid mobilization and oxidation, elevate plasma ketone body levels, and reduce tissue glucose uptake, thereby linking metabolism to improved cardiac function [47]. Correspondingly, the metabolic/myocardial fuel-supply hypothesis suggests that increased ketone body production from SGLT2is enhances energy supply efficiency to the heart, particularly beneficial under metabolic stress conditions like HF and diabetes [34]. The positive impact of SGLT2is on cardiac energetics and efficiency through ketone body production is highlighted by Subramanian et al. in patients with nonobstructive hypertrophic cardiomyopathy [49]. Additionally, Mantovani et al., in their comprehensive review, demonstrated the beneficial effects of SGLT2is on myocardial and liver fibrosis in individuals with non-alcoholic steatohepatitis and HF, irrespective of diabetes status [44]. Furthermore, Dong et al. provided evidence that SGLT2is also offer neurovascular protective benefits [48].

Obesity and type 2 DM contribute to HFpEF through the enlargement of visceral adipose tissue, which promotes inflammation and fibrosis in the heart. Enlarged epicardial adipose tissue restricts heart function and releases pro-inflammatory adipokines, increasing the risk of AF and impaired ventricular contraction. In their study, Tschöpe et al. showed that SGLT2is can reduce epicardial adipose tissue, lower cardiovascular risk, and potentially mitigate atrial fibrosis and serve as a treatment for AF and HF in obese HFpEF patients [43]. Also, epicardial adipose tissue around the atria is associated with a higher risk of AF. In type 2 DM and obesity, increased visceral fat creates a hypoxic and inflammatory environment, worsening HFpEF and promoting arrhythmias by disrupting electrical activity. The thickness of epicardial adipose tissue correlates with higher arrhythmogenesis and adverse outcomes [4].

Overall, SGLT2is enhance myocardial energy efficiency and decrease epicardial adipose tissue, which may help reduce atrial remodeling and lower the risk of AF in patients with obesity and type 2 DM.

#### 3.1.6. Sodium and Calcium Metabolism

During AF, excessive cytosolic calcium release from the sarcoplasmic reticulum activates calmodulin kinase type II (CaMKII) and ryanodine receptors, leading to increased cytosolic Ca^2+^. This overactivation of CaMKII and sodium/calcium exchangers results in delayed afterdepolarizations and spontaneous ectopic activity, contributing to stable AF circuits. CaMKII further supports AF by activating the calcineurin/nuclear factor of the activated T-cells (NFAT) pathway, which reduces L-type Ca^2+^ current and shortens action potential duration. DM exacerbates these processes by upregulating certain ion channels and altering gap junction proteins, thereby worsening arrhythmias and impairing cardiac function [4].

Chronic treatment with the dual SGLT-1&2 inhibitor sotagliflozin has demonstrated significant benefits in a rat model of HFpEF. Sotagliflozin improved left atrial cardiomyopathy by reducing cytosolic calcium ([Ca^2+^]) levels and enhancing mitochondrial calcium buffering, which mitigated mitochondrial swelling and decreased ROS production. These effects are linked to increased Na^+^/Ca^2+^ exchanger forward-mode activity, which alleviates cytosolic Ca^2+^ overload and reduces arrhythmia susceptibility. By normalizing mitochondrial function and improving calcium handling, sotagliflozin addresses the adverse effects of elevated cytosolic Ca^2+^ and ROS, making it a promising option for managing atrial remodeling and related arrhythmias in HFpEF [26].

In diabetic hearts, excessive sodium (Na⁺) influx through SGLT can lead to Na⁺ overload, contributing to arrhythmias and oxidative stress. SGLT2is such as empagliflozin counteract this by reducing Na⁺ influx and inhibiting the late sodium current (late-Iₙₐ), which is associated with arrhythmias and prolonged QT syndrome. Empagliflozin also improves calcium handling, mitochondrial function, and connexin expression, thereby enhancing cardiac function and reducing arrhythmias [3].

Empagliflozin’s protective effects against HF and related complications are partly due to its ability to reduce cytoplasmic sodium and calcium levels while increasing mitochondrial calcium. This likely results from the direct inhibition of the Na⁺/H⁺ exchanger [23]. Moreover, SGLT2is may mitigate cardiac injury, hypertrophy, fibrosis, and remodeling by directly inhibiting the myocardial Na^+^/H^+^ exchanger [34].

To summarize, SGLT2is, by modulating sodium and calcium metabolism, improve calcium handling and mitochondrial function, thus mitigating atrial remodeling and reducing arrhythmia risk in conditions like HF and DM.

#### 3.1.7. Other Mechanisms

##### Weight Loss

Obesity is associated with increased cardiac arrhythmias such as AF and sudden cardiac death, largely due to the abnormal electrical and structural changes in the heart linked to excess adiposity, as discussed earlier in this review [28,57]. SGLT2is effectively reduce body weight, fat mass, and visceral fat in patients with type 2 DM by promoting urinary glucose excretion and enhancing lipolysis through lower insulin and elevated glucagon levels [28]. Studies involving over 10,000 patients with type 2 DM have demonstrated that a weight loss of at least 5% achieved with SGLT2is correlates with a reduced risk of new-onset AF [3]. By decreasing excess adiposity and its adverse effects on cardiac structure and function, SGLT2is may reduce the risk of atrial remodeling and associated arrhythmias.

##### Hematocrit and Erythropoietin

The EMPA-REG OUTCOME trial indicates that SGLT2is such as dapagliflozin can enhance hematocrit levels and reduce cardiovascular events [20,28], potentially through their diuretic effects and influence on erythropoiesis. They achieve this by increasing erythropoietin production and inhibiting hepcidin, which regulates iron absorption and mobilization. In animal models, dapagliflozin’s cardioprotective effects appear to involve increasing erythropoietin levels and activating signaling pathways such as phosphorylated protein kinase B (p-Akt), phosphorylated Janus kinase 2 (p-JAK2), and phosphorylated mitogen-activated protein kinase (p-MAPK), which help reduce apoptosis. Thus, the therapeutic benefits of SGLT2is might be linked to their ability to elevate erythropoietin levels [28].

##### Blood Pressure

Previously published data indicate that SGLT2is reduce blood pressure by about 3.62/1.70 mmHg, primarily through initial fluid and sodium loss, followed by reductions in body fat and the inhibition of the renin–angiotensin–aldosterone system [20]. Additionally, a study of dapagliflozin shows a modest decrease in systolic blood pressure by 2.54 mmHg. The DAPA-HF trial confirmed that the observed benefits of the treatment are consistent across different blood pressure levels, although no significant interaction was detected (*p* = 0.78) [54]. Given that hypertension is a major risk factor for left atrial remodeling and AF, reducing blood pressure using SGLT2is may improve atrial remodeling and lead to better cardiovascular outcomes.

##### Angio- and Thrombogenesis

Atrial remodeling, marked by a granular and wrinkled endocardium with edema, fibrosis, and thrombotic aggregates, heightens the risk of thrombus formation in AF patients. This disruption in normal blood flow creates a pro-thrombotic state characterized by abnormal coagulation factors and increased platelet activation. DM worsens this condition by promoting inflammation, atrial myopathy, and epicardial adipose tissue expansion, which increases stroke risk through enhanced thrombus formation and altered coagulation [4].

Additionally, Fakih et al. suggest that components of the coagulation cascade produced by pathological endothelium contribute to atrial remodeling, creating a substrate for AF. Thrombin and Factor Xa (FXa) are implicated in maladaptive cardiac hypertrophy, inflammation, and fibrosis, influencing these processes beyond their roles in blood clotting. Targeting the angiotensin II type 1 receptor (AT1R)/NADPH oxidases/SGLT1/2 pathway could be a promising approach to prevent atrial remodeling and mitigate atrial cardiomyopathy in high-risk patients [57].

##### Molecules

Impaired myokine production and dysfunction within the muscle–adipose tissue axis lead to collagen accumulation in the heart, exacerbating atherosclerosis, endothelial dysfunction, oxidative stress, and mitochondrial dysfunction. These changes drive adverse cardiac remodeling, reduced angiogenesis, and muscle weakness, significantly affecting HF in type 2 DM. Myokines, which are produced by muscles, the heart, and adipose tissue, play a crucial role in regulating energy metabolism and inflammation. Some myokines, such as adropin, apelin, and irisin, offer protective effects, while others like myostatin can induce maladaptive changes. Adropin affects oxidative stress and autophagy through the lysosome-dependent pathway, a mechanism potentially targeted by SGLT2is. In their study, Berezin et al. found that in type 2 DM patients with HF, especially females, elevated adropin levels independently predicted improved hemodynamic performance with long-term treatment using the SGLT2i dapagliflozin, regardless of NT-proBNP levels [27]. Thus, SGLT2is may alleviate atrial remodeling and enhance cardiac function in type 2 DM by modulating myokines like adropin.

##### Epigenetic Effects

Recent translational studies have highlighted that SGLT2is may modulate atrial and ventricular remodeling by targeting epigenetic regulators involved in inflammation, oxidative stress, and profibrotic processes [28,38,39,42,50]. Notable effects include the downregulation of genes related to inflammatory responses, such as inflammasome mRNA [42,50], the improvement of mitochondrial function through the regulation of mRNA in adipocytes [38,39,42], and reductions in cardiac fibrosis by targeting collagen mRNAs [42,50]. 

## 4. Clinical Implications

### 4.1. Atrial Fibrillation

SGLT2is are already used as first-line treatments in all types of HF, their benefits being undeniable. Current data show their cardiovascular protection roles as positively influencing cardiac remodeling and consequently stopping the progression of clinical HF [9] or even regressing cardiac remodeling [14,50] in both diabetic and non-diabetic patients. Speculated mechanisms involved in this are their direct effect on profibrotic pathways [2], ventricular unloading and afterload reduction, and improvement in cardiac metabolism and energetics, among several other proposed mechanisms [36,47,54]. Moreover, recent translational studies highlight the potential benefits of SGLT2is in targeting mRNAs, which are well-established biomarkers for AF [28,38,39,42,50]. However, the role of SGLT2is in the current management strategies for patients with AF remains controversial.

The existing evidence, while not yet definitive, suggests a promising rationale for considering SGLT2is as a potential therapeutic approach for patients with AF in both diabetic and non-diabetic patients. Preclinical studies [20,21,26,38,39,50,57] indicate that SGLT2is may positively influence AF mechanisms through antifibrotic effects and improved electrical remodeling. While SGLT2is enhance cardiac bioenergetics by improving mitochondrial function and calcium homeostasis, their anti-arrhythmic potential is controversial [2,3,25,42,53,54].

SGLT2is have significantly impacted the prognosis of patients with HF according to the results of landmark trials on the topic [31,40,45,46,55,61]. Data from randomized clinical trials, including EMPA-REG, DECLARE-TIMI [1], DEFINE-HF, and PRESERVED-HF [8,9], along with several other studies [37,42,51,53], indicate a reduction in AF incidence and recurrence. Also, several other studies imply their potential efficacy in this regard [3,4,39,50]. These studies have collectively indicated that SGLT2is could be beneficial in managing AF through their molecular and cellular mechanisms, having a positive effect on both triggering and maintaining AF.

However, other research, including the CANVAS trial, did not consistently support this benefit. For instance, the CANVAS trial and additional studies [29,30] failed to show a significant reduction in AF associated with SGLT2is. The contrasting results from these studies underscore the complexity of AF and the multifaceted effects of SGLT2is.

Despite the limitations of current studies, which may not fully establish causality or the extent of the benefits, the observed effects of SGLT2is in related cardiovascular conditions provide a compelling basis for further investigation. The potential for SGLT2is to influence the progression of AF suggests that they could become an important component of comprehensive AF management strategies. Overall, while preliminary findings are promising, further research is needed to confirm the role of SGLT2is in AF.

### 4.2. Other Cardiac Conditions

SGLT2is are demonstrating promising results in various cardiac conditions beyond HF. Recent studies have explored the impact of SGLT2is following acute coronary syndrome. Lan et al. reported that in patients with both acute coronary syndrome and type 2 diabetes (T2D), adding empagliflozin to the standard acute coronary syndrome treatment at hospital discharge led to a reduction in left ventricular mass and improvements in diastolic function over a period of 3–6 months, compared to those with acute coronary syndrome and well-controlled type 2 DM who did not receive an SGLT2i [34]. Additionally, the ongoing EMPRESS-MI trial is expected to yield valuable data on the effects of empagliflozin through advanced cardiac and kidney imaging, as well as circulating biomarkers, in individuals at high risk of developing HF following acute myocardial infarction [56]. In a comprehensive review conducted by Patoulias et al., the authors evaluated the potential benefits of SGLT2is in acute HF. Their findings indicated that the current evidence is inadequate to support the use of SGLT2is in the treatment of acute HF [25]. SGLT2is have also been evaluated in patients with cancer therapy-related cardiac dysfunction, demonstrating improved outcomes in this population [53]. Lastly, in a separate study by Subramanian et al., the use of SGLT2is was shown to enhance diastolic function and functional capacity in patients with diabetes and nonobstructive hypertrophic cardiomyopathy with left ventricular function [49]. As new randomized trials continue to emerge, SGLT2is are expected to provide more comprehensive data, potentially expanding the indications to include a wider range of cardiac conditions, thereby broadening their therapeutic applications.

### 4.3. Other Non-Cardiac Conditions

Numerous emerging studies are exploring the potential benefits of SGLT2is beyond their established effects on cardiac conditions and diabetes [59]. Starting from their cardiovascular clearly proven benefits, Dong et al. studied the effects of SGLT2is on the central nervous system, suggesting neuroprotective actions [48]. Additionally, Mantovani et al., in their extensive review, highlighted the beneficial effects of SGLT2is on myocardial and liver fibrosis in individuals with non-alcoholic steatohepatitis and HF, regardless of their diabetes status [44]. However, additional prospective research is necessary to assess the efficacy of SGLT2is in these broader patient populations.

### 4.4. Cardiovascular Aging

Cardiovascular aging is one of the major risk factors associated with multi-morbidity and HF [24,44,54], particularly HFpEF. Key mechanisms in cardiovascular aging, which overlap with those involved in atrial and ventricular remodeling and targeted by SGLT2is, include inflammation, mitochondrial dysfunction, oxidative stress, and autophagy [24]. Additionally, in DM patients, advanced glycation endproducts (AGEs) accumulate during hyperglycemic states, promoting cardiovascular aging. Also, by binding to their receptors, AGEs promote inflammatory and oxidative responses, increasing the risk of AF development in these patients, thus showing a possible link with atrial remodeling [35]. Regarding vascular aging, recent data failed to show a major effect of SGLT2is [25]. Although current findings are promising, further large-scale studies are necessary to demonstrate a clear relationship between SGLT2is, atrial remodeling, and cardiovascular aging.

## 5. Conclusions and Future Perspectives

SGLT2si have emerged as powerful therapeutic agents that extend their benefits far beyond glucose control. The ability of SGLT2is to influence multiple molecular pathways involved in atrial remodeling presents significant implications for the treatment and prevention of AF and HF. This review underscores several key mechanisms through which SGLT2is contribute to mitigating atrial remodeling: reducing inflammation and oxidative stress, improving mitochondrial function, modulating autonomic and myocardial energy metabolism, and reducing fibrosis. Current European and American HF guidelines recommend SGLT2is as first-line treatment for HF patients, independent of their phenotype. Recent clinical and preclinical data support the multifaceted actions of SGLT2is, ranging from metabolic effects to molecular and cellular regulation, making them promising agents in altering the course of atrial remodeling. Further research is essential to better understand the clinical impact of SGLT2is on atrial remodeling in patients with AF and/or HF and to refine their therapeutic use for optimal patient outcomes.

## Figures and Tables

**Table 1:** Characteristics of the studies included in the review.

No.	Year	Study	Ref. No.	No. of Subjects	Subject Type	Comparison	Endpoints	Conclusions
1	2017	Persson F et al.	[29]	40.980	Human, type 2 diabetes	Dapagliflozin vs. DPP-4 inhibitors (dose not specified)	Major adverse cardiovascular events; non-fatal MI, non-fatal stroke, cardiovascular mortality, all-cause mortality, hospitalization for HF, atrial fibrillation, and severe hypoglycemia	Lower risk of major adverse cardiovascular events, hospitalization for HF and all-cause mortality
2	2017	Natali A et al.	[23]	75	Human, type 2 diabetes	Empagliflozin 10 mg/day vs. Satagliptin 100 mg/day	Global longitudinal strain, left ventricle ejection fraction, left atrial volume, E/E′, VO2max, cardiac autonomic function tests, and plasma biomarkers	
3	2018	David S et al.	[30]	review	v	GLP-1 agonists vs. SGLT2i vs. DPP-4 inhibitors	Structural and electrical atrial remodeling	GLP-1 agonists and SGLT2 and DPP-4 inhibitors do not accelerate or decelerate the development of new-onset AF
4	2018	David S et al.	[31]	review	Human	Several antidiabetic drugs	Reversal of cardiac remodeling	Metformin and SGLT2is can prevent or ameliorate HF
5	2019	Lee HC et al.	[20]	13	Spontaneous hypertensive rats	Empagliflozin 20 mg/day vs. control	Electrocardiography, echocardiography, invasive hemodynamic study, biomarkers, and tissue analyses	Beneficial effects on systemic blood pressure, renal function, ameliorated left atrial dilatation, intra-cardiac fibrosis, contraction, and relaxation dysfunction
6	2019	Iwakura K et al.	[32]	review	Human	Several antidiabetic drugs	Left ventricle hypertrophy, left atrium dilatation, systolic and diastolic function	SGLT2is decrease the incidence of hospitalization for HF, cardiovascular death, and left ventricle mass
7	2019	Ferrini M et al.	[22]	review	Human	Several antidiabetic drugs	Major adverse cardiovascular events, HF outcome, sudden cardiac death, AF	Glucose-lowering drugs may reduce the progression and avoid the onset of HF
8	2019	Shao Q et al.	[21]	96	Diabetic rats	Empagliflozin 10 mg/kg/day vs. empagliflozin 30 mg/kg/day vs. control	Left atrium diameter, interstitial fibrosis AF, atrial mitochondrial respiratory function, mitochondrial membrane potential, and mitochondrial biogenesis	Empagliflozin can ameliorate atrial structural and electrical remodeling and improve mitochondrial function and mitochondrial biogenesis in type 2 DM
9	2020	Kang S et al.	[33]	11	Cardiac fibroblasts from human atrial tissue	Empagliflozin 10/25 mg/day vs. control	Myofibroblast activity, cell morphology, and cell-mediated extracellular matrix remodeling	Empagliflozin significantly attenuated TGF-b1e-induced fibroblast activation
10	2020	Lan NSR et al.	[34]	44	Human, acute coronary syndrome, and type 2 diabetes	Empagliflozin 10/25 mg/day vs. control	E/e′ ratio, left ventricle mass index, and left ventricle diastolic function	Empagliflozin is associated with a reduction in left ventricle mass and favorable changes in diastolic function
11	2021	Lee TW et al.	[35]	review	Human	Several antidiabetic drugs	New-onset AF, atrial dilatation and fibrosis, mitochondrial function, and mitochondrial biogenesis	Empagliflozin and canagliflozin—no effect on the incidence of AF in patients with DM, dapagliflozin reduced AF risk and atrial flutter events
12	2021	Ibrahim NE et al.	[36]	review	Human	SGLT2is vs. control	Concentrations of atrial natriuretic peptide, B-type natriuretic peptide, and N-terminal pro-B-type natriuretic peptide, and reduction in high-sensitivity troponin	Reduction in concentrations of atrial natriuretic peptide, B-type natriuretic peptide, and N-terminal pro-B-type natriuretic peptide and high-sensitivity Troponin C in patients receiving SLGT2is
13	2021	Tanaka H et al.	[37]	210	Human, non-ischemic diabetic cardiomyopathy	SGLT2is vs. control	New-onset AF	SGLT2is can significantly reduce new-onset AF
14	2021	Nishinarita R et al.	[38]	12	Beagle dogs, induced AF	Canagliflozin (3 mg/kg/day) vs. control	Atrial effective refractory period, conduction velocity, and AF inducibility	Canagliflozin and possibly other SGLT2is might be useful for preventing AF and suppressing the promotion of atrial remodeling
15	2021	Bode D et al.	[26]	-	Obese rats	Dual SGLT-1&2 inhibitors (sotagliflozin 30 mg/day) vs. control	LA remodeling	Dual SGLT-1&2 inhibitors ameliorated left atrial remodeling and exerted an anti-arrhythmic effect on left atrial cardiomyocytes
16	2021	Lin YW et al.	[39]	32	Rats, induced left heart dilatation	Dapagliflozin 10 mg/kg/day vs. control	Electrocardiography and echocardiography, hemodynamic studies, and postmortem tissue analyses	Dapagliflozin suppresses cardiac fibrosis and endoplasmic reticulum stress
17	2021	Kearney A et al.	[40]	review	Human	SGLT2is	-	Reduction in left ventricle end-systolic volume, left ventricle end-diastolic volume, and N-terminal pro-B-type natriuretic peptide, suggesting reverse left ventricle remodeling
18	2021	Lee Z et al.	[24]	review	Human	SGLT2is	-	SGLT2is are beneficial for HF regardless of diabetic status
19	2021	Damy T et al.	[7]	review	Human	SGLT2is	-	SGLT2is are beneficial for HF regardless of diabetic status
20	2021	Kondo H et al.	[41]	364	Human	Canagliflozin 10 µmol/L (cell culture) vs control	Superoxide sources and the expression of inflammation, fibrosis, and myocardial stretch genes	Canagliflozin has global anti-inflammatory and anti-apoptotic effects in the human myocardium
21	2021	Vrachatis DA et al.	[42]	review	Human	SGLT2is	-	Pathophysiologic mechanisms involved in AF seem to be favorably affected by SGLT2 inhibition
22	2021	Tschope C et al.	[43]	review	Human	Several antidiabetic drugs	-	SGLT2is are beneficial for HF regardless of diabetic status and LVEF
23	2022	Mantovani A et al.	[44]	review	Human	Several antidiabetic drugs	-	SGLT2is reduce major adverse cardiovascular events, all-cause mortality, and hospitalization for HF regardless of the presence or absence of type 2 DM and left ventricle ejection fraction
24	2022	Von Lewinski D et al.	[2]	402	Human, HF, with reduced or midrange ejection fraction	Ertugliflozin 5 mg/day vs. control	Total burden of ventricular arrhythmias	Trial design
25	2023	Manolis AA et al.	[3]	review	Human	SGLT2is	-	SGLT2is can reverse atrial and ventricular remodeling and ameliorate mitochondrial dysfunction
26	2022	Savage P et al.	[45]	review	Human	SGLT2is	-	SGLT2is are associated with a greater incidence of clinical benefits (as defined by rates of death, HF events, time to first HF event)
27	2022	Theofilis P et al.	[46]	review	Human	SGLT2is	-	SGLT2ihave beneficial effects on cardiac remodeling and attenuate HF progression
28	2022	Thirumathyam R et al.	[47]	20	Human, type 2 diabetes, body mass index ≥ 28 kg/m^2^, glycated hemoglobin ≤ 9%, fasting C-peptide > 500 pmol/L	Empagliflozin 25 mg/day vs. insulin	Change in myocardial diastolic function	Study protocol
29	2022	Dong M et al.	[48]	review	Human	SGLT2is vs. GLP-1 agonists	-	GLP-1RAs and SGLT2is cross the blood–brain barrier and can be applied in the treatment of central nervous system diseases
30	20222	Subramanian M et al.	[49]	48	Human, nonobstructive hypertrophic cardiomyopathy	Empagliflozin 10/25 mg/day or dapagliflozin 5/10 mg/day vs. control	Improvement of at least 1.5 in the E/e ratio and a reduction of >1 NYHA functional class	SGLT2is have favorable effects on diastolic function and functional capacity
31	2022	Patoulias D et al.	[25]	review	Human	SGLT2is	Role of SGLT2i in acute HF	Insufficient data to substantiate the use of SGLT2is in acute HF
32	2023	Koizumi T et al.	[50]	48	Diabetic rats	Empagliflozin 30 mg/kg/day vs. control	Examine whether empagliflozin suppresses mitochondrial-ROS generation and mitigates fibrosis.	Empagliflozin reduced mitochondrial oxidative stress and prevented atrial remodeling in a murine model of type 2 DM
33	2023	Berezin AA et al.	[27]	417	Human, HF with preserved, midrange, and reduced ejection fraction	Dapagliflozin 10 mg/day vs. control	Modulation of adropin levels	The levels of adropin seem to be a predictor for the favorable modification of hemodynamic performances during SGLT2 inhibition
34	2023	Chen Y et al.	[28]	review	Human	SGLT2i	Molecular mechanisms of SGLT2i on ventricular remodeling	SGLT2 receptor is almost not expressed in the heart, so its target is difficult to determine at present
35	2023	Lorenzo-Almorós A et al.	[4]	review	Human	Several antidiabetic drugs	-	Structural, electrical, and autonomic remodeling may lead to AF
36	2023	Nassif ME et al.	[51]	587	Human, HF with preserved or reduced ejection fraction	Dapagliflozin 10 mg vs. control	Change in the Kansas City Cardiomyopathy Questionnaire Clinical Summary Score at 12 weeks	Dapagliflozin improved Kansas City Cardiomyopathy Questionnaire Clinical Summary Score at 12 weeks by 5.0 points
37	2023	Scatularo CE et al.	[52]	54	Human, endomyocardial fibrosis	-	Demographic, clinical, biochemical, and imaging variables	7.4% of patients with endomyocardiofibrosis were treated with SGLT2is
38	2024	Avula V et al.	[53]	1280	Human, cancer therapy-related cardiac dysfunction	SGLT2is vs. control	Acute HF exacerbation, All-cause mortality	Patients on SGLT2is had a lower risk of acute HF exacerbation
39	2023	Escobar C et al.	[54]	review	Human	Dapagliflozin 10 mg/day	-	Dapagliflozin has effects on reversing cardiac remodeling, reducing cardiac fibrosis and inflammation, and improving endothelial dysfunction
40	2024	El-Saied SB et al.	[55]	70	Human, HF with mildly reduced ejection fraction, and DM	Empagliflozin 10 mg/day or dapagliflozin 10 mg/day vs. control	Demographic, clinical, biochemical, and imaging variables	SGLT2is cause significant improvement of left atrium volume and functions, with further improvement of left ventricle diastolic and longitudinal functions
41	2024	Carberry J et al.	[56]	100	Human, left ventricular systolic dysfunction after myocardial infarction	Empagliflozin 10 mg/day vs. control	Change in left ventricle end-systolic volume indexed to body surface area over 24 weeks from randomization	Study design
42	2024	Fakih W et al.	[57]	20	Human and porcine model	SGLT2i	Examined whether activated factor X induces pro-remodeling and profibrotic responses in atrial endothelial cells	Sotagliflozin and empagliflozin prevented the FXa-induced eNOS-NO/ROS imbalance and the induction of endothelial senescence
43	2024	Žlahtič T et al.	[58]	case report	Human	-	-	SGLT2is are a cornerstone in the management of HF patients
44	2024	Kodur N et al.	[59]	review	Human	-	-	SGLT2is have shown promise in reducing HF-related morbidity and mortality

AF, atrial fibrillation; DM, diabetes mellitus; DPP-4, dipeptidyl peptidase-4; GLP-1, glucagon-like peptide 4; SGLT2i, sodium–glucose cotransporter 2 inhibitor; HF, heart failure; TGF, transforming growth factor; ROS, reactive oxygen species.
